# Ethical Challenges Posed by Advanced Veterinary Care in Companion Animal Veterinary Practice

**DOI:** 10.3390/ani11113010

**Published:** 2021-10-20

**Authors:** Anne Quain, Michael P. Ward, Siobhan Mullan

**Affiliations:** 1 Sydney School of Veterinary Science, Faculty of Science, University of Sydney, Sydney, NSW 2006, Australia; michael.ward@sydney.edu.au; 2 School of Veterinary Medicine, University College Dublin, Belfield, Dublin 4, Ireland; siobhan.mullan@ucd.ie

**Keywords:** advanced veterinary care, standard of care, companion animals, veterinary ethics, conflict of interest, quality of life, dysthanasia

## Abstract

**Simple Summary:**

Veterinary care of companion animals, particularly dogs and cats, continues to advance, with some companion animals receiving a standard of care equal to or exceeding that of human patients. While this has the potential to improve animal welfare and benefit other stakeholders, including animal owners and veterinary team members, it also poses ethical challenges. We discuss key ethical challenges associated with AVC, including its relationship to standards of veterinary care, its potential to perpetuate poor quality of life and suffering, cost and accessibility of veterinary care, conflicts of interest, and concerns about experimentation without appropriate ethical review. We conclude by suggesting some strategies for veterinary teams and other stakeholders, such as professional bodies and regulators, to address these concerns.

**Abstract:**

Advanced veterinary care (AVC) of companion animals may yield improved clinical outcomes, improved animal welfare, improved satisfaction of veterinary clients, improved satisfaction of veterinary team members, and increased practice profitability. However, it also raises ethical challenges. Yet, what counts as AVC is difficult to pinpoint due to continuing advancements. We discuss some of the challenges in defining advanced veterinary care (AVC), particularly in relation to a standard of care (SOC). We then review key ethical challenges associated with AVC that have been identified in the veterinary ethics literature, including poor quality of life, dysthanasia and caregiver burden, financial cost and accessibility of veterinary care, conflicts of interest, and the absence of ethical review for some patients undergoing AVC. We suggest some strategies to address these concerns, including prospective ethical review utilising ethical frameworks and decision-making tools, the setting of humane end points, the role of regulatory bodies in limiting acceptable procedures, and the normalisation of quality-of-life scoring. We also suggest a role for retrospective ethical review in the form of ethics rounds and clinical auditing. Our discussion reenforces the need for a spectrum of veterinary care for companion animals.

## 1. Introduction

The first half of the twentieth century saw the key focus of veterinary practice pivot from equine patients and livestock, as sources of transport, food, and fibre, to dogs and cats, primarily for companionship. According to Gardiner, dogs, and later cats, were reframed not only as legitimate veterinary patients, but as “suitable recipient[s] for a new type of scientifically driven veterinary medicine, where cost was not always a limiting factor in deciding upon treatment, as it was with livestock” [1].

According to The CALLISTO Project, the term “companion animal” refers to “domesticated, domestic-bred or wild caught animals, permanently living in a community and kept by people for company, amusement, work (e.g., support for blind or deaf people, police or military dogs) or psychological support—including dogs, cats, horses, rabbits, ferrets, guinea pigs, reptiles, amphibians, birds and ornamental fish” [2]. Although large animals such as horses and cattle can and do fulfil the role of companions, companion animal practice, sometimes referred to as small animal practice, tends to focus on dogs, cats and other small companion animals [3]. For the purposes of this discussion, the term “companion animal” will refer to the latter.

As companion animals were increasingly considered family members [4], companion animal practice borrowed methods and values from human medicine. According to Knesl and colleagues, “the strengthening of the bond between humans and their pets has changed the landscape for veterinary medicine, with highly bonded owners showing an increasing willingness to do whatever it takes to maintain the health of their animals” [5]. To this end, it could be said that companion animal practice has co-evolved with the human–companion animal bond. People are spending more time with, and more money on, caring for companion animals [6]. In developed countries, companion animals can receive a standard of healthcare similar to or at times exceeding that available to humans [7].

In this paper, we explore ethical challenges posed by the advanced veterinary care of companion animals. However, first, it is important to explore what we mean by advanced veterinary care.

## 2. What Constitutes Advanced Veterinary Care?

The Oxford English Dictionary defines “advance” as “a step forward, a degree of progress actually accomplished; a development; an improvement” [8]. The history of veterinary science is a history of advances in scientific knowledge and its practical application. The term “advanced” is defined as “far on or ahead in any course of development; (hence) progressive, ahead of one’s time” [9]. What is ahead of one’s time now may subsequently become the acceptable standard, or even below the standard in the future. Because clinical veterinary care is continually advancing, there is no fixed definition of what constitutes advanced veterinary care (AVC), as it would rapidly become outdated [10]. Yet, the term is commonly used by veterinarians and others to refer to a particular type of veterinary care.

Advances and AVC are motivated by a drive to improve the quality of care, though what is meant by improved quality of care in the veterinary sector is not easily defined [11]. For example, it may be assessed according to improved animal welfare, improved clinical outcomes, improved client satisfaction, or even other factors such as practice profitability.

Conceptually, AVC has been positioned as being at one end of a continuum or spectrum of acceptable care, with basic veterinary care situated at the other end of the spectrum [12]. According to this model, basic veterinary care is characterised by low costs, low technology, basic skills and being less resource dependent, while AVC is characterised by higher costs, advanced skills, state-of-the-art techniques and equipment, and being more resource dependent [13]. However, proponents of this view add that this spectrum does not imply that AVC is “better”, “acceptable”, “successful”, “standard of care”, a “product of practice experience” or “more challenging” when compared with care along that spectrum [13]. AVC is considered as going beyond the “standard of care” at which general practitioners are expected to practice.

Standard of care (SOC), also known as standard of practice, has been defined as that required of and practiced by the average, reasonably prudent and competent veterinarian [12,14]. The SOC is referred to in codes of conduct. For example, the Veterinary Practitioner’s Code of Professional Conduct in New South Wales states that, in addition to animal welfare, “the basic principles of professional conduct for a veterinary practitioner are…the maintenance of professional standards to the standard expected by: (i) other veterinary practitioners, and (ii) users of veterinary services, and (iii) the public” [15]. It further requires that veterinarians “must maintain knowledge to the current standards of the practice of veterinary science in the areas of veterinary science relevant to his or her practice” and ensure that they practice according to current standards[15].

The SOC may vary according to the context in which the veterinarian is practicing [14,16]. Like AVC, it is difficult to determine exactly what constitutes SOC, as this too is evolving [14]. In human medicine, SOC has been defined through landmark legal cases, evidence-based clinical practice guidelines, and statutes defining medical malpractice [17]. In the era of evidence-based veterinary medicine (EBVM), it is important that the SOC changes in light of new knowledge, skills, and technology, underpinned by good quality evidence [18].

In order to characterise AVC, a literature search on Web of Science Databases (incorporating Web of Science Core Collections, CABI: Cab Abstracts, MEDLINE, Current Contents Connect, BIOSIS Previews, Zoological Record and SciELO Citation Index) was undertaken using the terms “advanced care” and “dog” or “cat”, limited to the subject area “veterinary sciences”. The search was limited to a five-year period (6 September 2016 to 6 September 2021) and yielded 127 journal articles. Of these, 20 focused on intensive care unit patients, 16 on advances in treatment, nine on advances in diagnostics, and two on advances in data collection. An additional three papers reviewed advances over periods ranging from 40–100 years, and the remainder were irrelevant (not focused on companion animals; focused on laboratory animals or in vitro studies only; or discussed animals in an advanced disease state or of an advanced age).

The term “advanced” has been most commonly used in the context of advanced imaging, where modalities including ultrasound, computed tomography (CT) and magnetic resonance imaging (MRI) have been considered advanced in relation to conventional (film or digital) radiography and planar scintigraphy [19]. Advanced imaging offers the possibility of more accurate diagnosis and staging than conventional imaging, and increased sensitivity and increased diagnostic confidence, with the potential for improved patient outcomes. However, increased sensitivity may increase the prevalence of incidental imaging findings in asymptomatic patients, or symptomatic patients undergoing imaging for another reason, referred to as “incidentalomas” [20]. These pose ethical challenges in both human and veterinary medicine, including how to communicate such findings to patients or clients, and whether further diagnostics or treatments should be performed [20,21]. Indeed, “overdiagnosis” is a recognised problem associated with increasingly sensitive diagnostic testing for conditions such as breast cancer in human patients, leading to treatment that does not benefit and may even harm the patient (“overtreatment”) [22]. It has been also argued that over-reliance on advanced imaging may lead to a reduction in the history taking and physical examination skills of veterinarians, leading to an inflation of veterinary costs [19].

In addition to advanced imaging, in recent discussions of veterinary ethics, AVC has been associated with cancer chemotherapy, radiotherapy, immunotherapy, stem-cell treatment, personalised medicine, hip arthroplasty, heart-valve replacement, dialysis, 3-D printing of prostheses, interventional radiology, stereotactic radiosurgery, interventional cardiology and surgical innovation [23,24,25,26,27,28]. However, what is considered to be AVC may vary between practitioners: a procedure that is considered routine by a veterinary surgical oncologist may be considered advanced by a general practitioner.

In many countries, AVC in companion animals is generally provided by veterinary specialists. As AVC has become more common, there has been a rapid increase in veterinary specialists. For example, in the US, the number of veterinarians working in referral or specialty practice increased by 98.4% between 2008 and 2013, and by 49.1% from 2013–2018 [29]. According to Tannenbaum, specialists “…provide advanced services that are not within the province of ordinarily competent generalists”[16]. Furthermore, they must perform to a higher standard of competence than general practitioners [16].

A number of commentators have suggested the potential unintended consequence of AVC in reducing the spectrum of care offered by general practitioners [13,30]. For example, veterinary students are commonly taught surgical skills by surgical specialists in teaching hospitals, with access to sophisticated, expensive diagnostic imaging and treatment modalities. Patients seen in such facilities are more likely to have uncommon or complex conditions. Furthermore, those admitted to university teaching hospitals as emergency patients tend to be referred internally to specialist services, under “an assumption that owners will want to pursue expensive diagnostic testing and advanced treatment for their pets” [12]. As a result, according to Stull and colleagues, “veterinarians (most notably recent graduates) may be unaware of, and lack the knowledge and skills to offer, a wide spectrum of care options for a given condition and therefore may be unable to communicate to clients the relative effectiveness and costs of options along this spectrum” [30].

Concerningly, AVC may be conflated with SOC, leaving practitioners and those who do not practice AVC exposed to charges of incompetence or negligence, or the risk of professional liability [14]. As a result, some veterinarians may practice AVC defensively, to reduce the risk of complaints and liability [12]. Even specialists are not immune to such influences. An investigation of the impact of client complaints on small animal veterinary internists found that just over 70% changed the way they practiced medicine due to fear of a client complaint, and 80% agreed to perform treatment requested by an owner even where they did not feel it was medically necessary [31]. Around 35% performed invasive procedures, against their professional judgement, to avoid a client complaint, where the owner demanded this treatment [31]. According to Rosoff and colleagues, “the skills to carry out technologically sophisticated diagnostic and therapeutic actions—in many ways mirroring those routinely employed in human medicine—have developed within a culture and professional standard of yielding to what clients (i.e., owners) want to have done to and for their animals” [28].

For the purposes of this discussion, AVC will be defined as veterinary care that exceeds the current SOC, including veterinary intensive care, which is typically—but not exclusively—provided by specialists.

## 3. Poor Quality of Life, Dysthanasia and Caregiver Burden

While AVC may be driven by a desire to improve clinical outcomes, for example, by curing or managing disease, and may achieve this, this is not the inevitable or only possible end result. Broadly speaking, any veterinary intervention may lead to better, unchanged or worsened patient quality of life (QOL). Ethical concerns have been raised regarding AVC where it leads to unchanged or worsened patient QOL: the potential to prolong the life of the patient, despite poor or declining animal welfare. Furthermore, in providing such care in some situations, there is a risk that veterinary team members become complicit in animal suffering.

The term “dysthanasia” (from the Greek *dys*, for difficult, and *thanatos*, referring to death) has been used to describe death associated with “excessive treatment in relation to the clinical condition and its expected prognosis” [32]. In medicine, discussions around dysthanasia have largely focused on intensive care units, where treatments that have the potential to delay death such as artificial ventilation, haemodialysis, parenteral nutrition, the use of drugs (particularly vasoactive sympathomimetic amines) and resuscitation are common [32]. While these measures may provide vital supportive care in the face of acute illness, it is their use in patients who are unlikely to recover that has been criticised, as they may lead to people dying in ways that conflict with their expressed preferences [33]. Similarly, some interventions in veterinary intensive care may be viewed by veterinary team members as being at odds with the perceived interests of the patient [34,35]. An example is cardiopulmonary resuscitation (CPR) of elderly animals, or those with terminal illnesses. While euthanasia is a major confounding factor in assessing CPR outcomes in dogs and cats, outcomes of resuscitation are poor. In a prospective study of 172 dogs and 47 cats administered CPR following cardiac arrest, 7% of dogs and 19% of cats survived to hospital discharge [36]. Unlike human patients, who may document their wishes via an advanced care directives to avoid dysthanasia, companion animals cannot opt out of heroic life-prolonging treatment.

Provision of AVC may be considered futile or non-beneficial in some cases and may lead veterinary team members to experience moral distress. In a survey of UK veterinarians (n = 58), a client wishing to pursue treatment despite poor animal welfare was rated as the most stressful ethical dilemma [37]. A survey of 889 veterinarians in North America found that 57% sometimes and 22% often managed cases where an animal owner requested treatment that the veterinarian considered to be futile [38]. In the same study, 51% of respondents reported refusing to provide what they considered to be futile treatment. A survey of 183 veterinary anaesthetists found that 63% were concerned that veterinary interventions were associated with animal suffering, and 18% reported that euthanasia was delayed “beyond the point the anaesthetist felt was appropriate” [39].

Complicating decisions to continue treatment despite poor QOL is a lack of consensus around what constitutes a minimal QOL for companion animals, and what constitutes acceptable morbidity risk. For example, a retrospective study of eight cats with oral neoplasia treated with radical mandibulectomy (removal of 75–90% of the mandible) [40] sparked a debate regarding the ethical justification of the intervention. A veterinary specialist criticised the authors’ conclusion that the treatment should be considered given high morbidity and mortality rates reported in the case series [41]. The authors defended their recommendation on the grounds that removal of lytic tumours in animals removes a source of profound pain and thus improves QOL [42]. The outcomes evaluated by the original study included survival, local disease recurrence, metastasis, whether cats could meet nutritional requirements with oral feeding, and owner satisfaction, but did not incorporate QOL assessment or overall welfare scoring, nor was this required by the journal.

Owners and clinicians may be biased in their assessment of outcomes in which they have invested emotional, financial or technical resources. The use of appropriately constructed, validated, multi-dimensional QOL scoring tools at multiple time points may help to minimise this bias by ensuring all relevant factors are considered [43]. In the above case, objective assessment of QOL may have aided this discussion, but the study was retrospective. Therefore, while it would be ideal for journals to require authors to include QOL assessment when investigating outcomes of an intervention, this should be underpinned by access to validated QOL scoring tools [44,45], widespread availability of training on how to use them, and their routine use in veterinary practice.

Tannenbaum argued that the drive to provide AVC may be difficult to resist for those with the ability to do so. He wrote that “specialists are trained, they *exist*, to provide advanced procedures. A difficult medical case cannot only excite a specialist’s intellectual curiosity (there is nothing wrong with this) but also provide a challenge to the specialist’s acumen—perhaps even to the state of knowledge of the speciality itself. Thus, specialists can have a professional interest in solving a problem rather than ‘giving up’ with euthanasia”[16]. It should be noted that a desire to intervene and prolong life is not necessarily exclusive to specialists. More recently, Taylor observed that “a question being increasingly asked is whether there are many clinicians who currently view euthanasia as a failure rather than a considered, considerate option for a struggling animal” [46].

Furthermore, the desire to prolong life may be driven by owners, who may seek out practitioners offering AVC because they wish to avoid euthanasia or ensure they have done everything in their power to save their companion animal. As a pioneer in the study of the human–companion animal bond [4], Serpell argues that strong anthropomorphic attachments to companion animals may lead owners to pursue prolonging the life of an animal, even if that animal has a terminal illness and is suffering [47]. He argues that such tendencies have been facilitated by AVC and “the increasing availability of previously inaccessible treatment options” [47]. Depictions of veterinary care in the media and television may fuel unrealistic client expectations [46]. Given that the majority of veterinarians are current or former pet owners with “histories of strong emotional attachments to companion animals” [48], Serpell questions their ability to achieve “sufficient psychological distance” to permit an unbiased assessment of their welfare [47].

However, even in situations where veterinarians disagree with an owner’s desire to pursue what the veterinarian may consider to be futile treatment, in most jurisdictions in the world, animals are considered the property of the owner under law. Owners may have a right to refuse euthanasia, even where death is imminent. In such cases, veterinarians may be forced to continue to treat an animal, even where they feel it is against the animal’s best interests. As in the field of medicine, there is a dearth of evidence indicating when treatment is, beyond reasonable doubt, futile [32]. Unlike laboratory animals, the veterinary treatment of companion animals is not limited by pre-determined humane endpoints. This may lead to a situation where a beloved companion animal, under the care of a veterinary team, may suffer more at the end of their lives than a laboratory animal undergoing an experiment with ethics committee oversight, where humane endpoints are predetermined [49].

It has been argued that the availability of AVC incorporating aspects of home care for animals with chronic conditions may increase caregiver burden among companion animal owners [50]. Caregiver burden is described as distress associated with the emotional, financial and practical demands of caring for a patient [51]. Owners caring for chronically ill dogs described impacts on multiple dimensions of their lives, including changes in the use of their homes, changes in working schedule, altered routines, medication regimes and increased veterinary visits [50]. While some owners find a sense of purpose in increased caring responsibilities, around half of owners looking after seriously ill companion animals reported high levels of burden [51]. While this burden was lessened with the knowledge that euthanasia could be chosen for an animal, this choice was experienced as an added burden by some owners [50].

## 4. Financial Cost and Accessibility of Veterinary Care

Unless subsidised, for example by a charity, as part of a clinical trial or for teaching purposes, the cost of veterinary care is borne entirely by companion animal owners. Due to its association with sophisticated, newer technology and application by specialists, AVC is associated with higher costs. This raises ethical questions around equity, as AVC is not accessible to the majority of veterinary patients.

The costs of AVC occur in the context of rising costs of veterinary care in general, particularly for companion animals [52]. Cost is a recognised barrier to accessing to veterinary care, as it is in human health care. In order to remain sustainable and make a profit, service providers must charge clients for products, services and time. Overheads include equipment costs, staffing (higher in facilities providing specialist and 24-hour care), other operational costs, insurance, training and professional development. Rising costs may be due in part to an increase in SOC. In addition, veterinary students graduate with significant debt, and require higher wages to service these debts [53,54]. It has been suggested that increased costs in veterinary care in general may be related to an increase in veterinary student tuition fees over the last two decades [53].

In bioethics, the question of access to healthcare falls under the principle of justice, insofar as it relates to questions of fairness, entitlement, and equitable distribution of healthcare resources [55]. Ideally, healthcare should be universal, continuous and affordable for patients and clients; however, due to costs and other barriers (for example access to transport), veterinary care is not accessible to all companion animal owners. There is no “safety net” to ensure that all companion animals receive required care [28].

In a survey of US dog and cat owners, 40% reported that cost prevented them from seeking veterinary care in the past five years [56]. According to the Access to Veterinary Care Coalition, over 29 million companion animals live in households participating in the Supplemental Nutritional Assistance Program, with millions more living in households that are financially struggling [57]. Additionally, veterinary care may simply not be available in some underserved communities. Barriers to veterinary care impact animal welfare, as well as the experiences of animal owners and veterinary team members [58].

Surveys of ethical challenges encountered by veterinary team members consistently identify client financial limitations as the most common [37,59,60] or one of the most common [39,61,62] ethical challenges encountered. These studies do not discuss potential moral distress experienced by companion animal owners who wish to pursue treatment but are not in a financial position to do so. Higher costs associated with AVC are likely to pose a barrier to access to AVC. This means that a large proportion of owners cannot fund the care their animals need, while a smaller proportion of owners can pay for AVC—even when this care is not required or recommended [28].

It could be argued that this really is not a problem with AVC. After all, all veterinary medicine, including AVC, is a form of private medicine, paid for by clients whose funds would otherwise not necessarily be spent treating animals *not* owned by those clients [7]. However, AVC is associated with increased costs in veterinary medicine, both directly as a result of costs incurred through provision of advanced care itself, and indirectly, through raising the overall SOC. An unintended consequence of this is an increase in the overall cost of pet ownership [63], and a reduction in the accessibility of veterinary care.

Furthermore, it may not be appropriate that the veterinary market dictates what types of treatment are available. Where it does, it is possible that owners with access to substantial funds may pursue “highly interventional” medicine and surgery [7].

As with all veterinary interventions, AVC may lead to increased costs to clients when unnecessary diagnostics and treatments are carried out. “Overutilisation” or “overservicing” subject animals to unnecessary discomfort, while also subjecting clients to unnecessary costs [16,20].

Strong attachment to animals may influence an owner’s willingness to incur higher veterinary fees [64]. In a survey of 50,000 Canadian households, Ipsos-Reid segmented pet owners into four categories: pet humanists (31%); pet pleasers (25%); conscientious pet lovers (24%); and pet traditionalists (20%) [65]. Pet humanists were defined as caring and devoted pet owners, and despite having the second highest level of income (when compared with conscientious pet lovers), were the highest consumers of veterinary services. They may feel guilty for declining treatment for financial reasons. According to the report, “if their pet developed a chronic disease a full 41 percent [of pet humanists] would spend $1000 or more trying to aid in its recovery and 85 per cent would go into debt if necessary to provide for the pet’s well-being” [65]. The authors added that “the loyalty and devotion of these people to their pet is attractive…the pet humanists segment is, therefore, the primary target for the pet food and pet service market” [65].

Pet humanists were also the most likely owners to insure their pets. Pet insurance may reduce “economic euthanasia” [66,67,68], benefitting animals, their clients and veterinary team members who may otherwise experience moral distress. However, pet insurance premiums may not be affordable for many pet owners. It is possible that the growth of pet insurance has been driven by increased costs associated with AVC [69]. At the same time, increased costs of veterinary care drive up the cost of pet insurance premiums [46].

An alternative means of paying veterinary bills is crowdfunding. Platforms such as GoFundMe promote themselves to pet owners for this purpose [70], while others such as CoFundMyPet encourage veterinarians to promote crowdfunding to their clients [71]. While crowdfunding may facilitate payment of veterinary fees, it may facilitate provision of “extreme” interventions [72]. While it may solve ethical challenges related to finance, crowdfunding presents additional ethical challenges for multiple stakeholders—including fundraisers, funders, platforms and regulators [73]. For example, crowdfunding may be undertaken by those with the means to pay veterinary bills [74], or to defraud funders [75,76].

## 5. Conflicts of Interest

Tannenbaum described conflicts of interest as “the basic fact of veterinary ethics”, primarily because of the potential conflict between the interests of the client and those of the animal [16]. He expanded that “time and again, veterinarians are thrust into the middle of these conflicts, wishing to satisfy the needs of both patient and client but unable to do so.” [16]. Conflicts between the interests of the owner and the interests of the animal are one of the most common ethical challenges faced by veterinarians and veterinary team members [37,38,59,60,61,62]. Yet, it is possible for veterinarians, like other healthcare professionals, to be influenced by considerations other than the needs of the client and the patient.

Like other professionals, veterinarians earn an income for their work, and may have a financial interest in performing AVC and higher cost interventions. This creates a potential conflict of interest [28]. For example, a veterinarian may recommend a particular treatment because they, or the practice, will earn more, rather than because that particular treatment is in the animal’s best interests. According to Rosoff and colleagues, “even those vets on salary, like their human medical counterparts, are well aware of the necessity to generate sufficient income to support themselves and their institutions. Hence, there is an inherent conflict of interest that may underpin their recommendations and could lead to overtreatment or inappropriate treatment” [28].

As mentioned previously, professional development may create a potential conflict of interest. In a study of companion animal veterinarians in Austria, the use of new technologies and techniques were correlated with veterinarians’ desire for self-improvement, and identified as a source of motivation in working life [77]. Springer and colleagues hypothesised the existence of four ethical decision orientations utilised by companion animal veterinarians when managing ethically challenging situations. One of these, “development oriented”, prioritises a veterinarian’s own desires to advance veterinary practice [69]. Development-oriented veterinarians agreed more strongly with the statements that “veterinary medicine should offer the same diagnostic options as human medicine”; “it is important to promote the advancement of small animal medicine for future patients” and “it is important for the veterinary profession to keep developing innovative methods, even though it is impossible to predict possible complications” [69]. While attitudes to the rapid development of diagnostic and treatment options varied between countries (for example, UK veterinarians were less development oriented than those in Denmark or Austria), this may reflect other factors, such as being more accustomed to mandated continuing professional development (CPD) [69]. Less experienced and younger veterinarians were more likely to be development oriented. This may reflect their relatively recent training and exposure to AVC in university teaching hospitals [69], or other factors such as a less nuanced understanding of standard of care, inability to predict unintended consequences of AVC, or a combination of these. It is possible that the development-oriented veterinarian may prioritise AVC over the interests of their patient.

Credentialling requirements may create a conflict of interest for veterinarians in training. For example, veterinarians specialising in dentistry must meet minimum required case-log quotas, and therefore “may be tempted to perform orthodontic procedures on questionable cases for the sole purpose of meeting the quota requirement” [78]. Alternatively, AVC interventions may be performed by an already credentialled specialist to publicise or market their skills and services, for example on social media, in newsletters or publications, or even on television.

Another conflict of interest may occur when the treatment of a patient becomes a clinical trial. In such an instance, the veterinarian’s interest in testing a hypothesis (for example, that treatment X will result in therapeutic outcome Y) may be in conflict with the interests of the patient (for example, to receive a different treatment, or no treatment at all) [16]. We will discuss this further in Section 6.

## 6. When Does Advanced Veterinary Care Become Experimentation?

According to Verstraete and Tannenbaum, one of the core principles common to veterinary codes of ethics is that “veterinarians should base diagnoses and treatments on the best available scientific knowledge, and should not employ techniques of which the efficacy and safety have not been established by sufficient scientific evidence” [78].

The first part of this statement promotes a development-oriented approach, requiring veterinarians to stay abreast of the “best available scientific knowledge”, while setting limits on its application. Notably, it should be applied only when the safety or efficacy of a technique have been established. However, the safety and efficacy of novel or innovative techniques may not have been established before they are used on veterinary clinical patients. What amounts to “sufficient scientific evidence” is open to interpretation. There may be scant evidence supporting innovative diagnostic and treatment modalities and techniques.

In a letter to the *Veterinary Record* in 2017, eleven veterinary specialists raised concerns about what they perceived as a “progressive loss of clarity between acts of veterinary surgery and animal experimentation, particularly with respect to companion animals” [27]. They argued that while experimental treatment on laboratory animals is overseen by ethics committees, the need for such oversight in companion animal practice was potentially greater due to the potential influence of competing interests of animals, animal owners, veterinarians and other parties such as sponsors.

Such concerns are the basis of “EthicsFirst”, “a group of veterinary and non-veterinary professionals who share common concerns about related areas of companion animal clinical practice in which boundaries are being pushed to extremes” [72]. These include “unproven interventions” and “unregulated research”. Through publications and presentations, EthicsFirst seeks to promote “independent and prospective ethical review” of “extreme” interventions, in addition to prioritisation of animal welfare [72].

The use of novel or innovative treatments in veterinary practice is less regulated than it is for medical procedures [79]. There may be a lack of consensus on what counts as “novel”, “innovative”, or indeed “experimental” in a clinical setting, particularly a setting where empirical treatment trials on individual patients are common due to cost constraints [59]. In the United Kingdom, the Veterinary Surgeons Act (1966) states that “the clinical investigation and management of the health of animals is generally considered to be recognised veterinary practice when it involves an intervention which is of direct benefit to the animal or its immediate peer group” [80]. There is a danger of “selective interpretation” of what constitutes “recognised veterinary practice” [72]. Additionally, veterinarians who work in clinical practice may have limited exposure to ethics committees and may struggle to identify instances where there is a need for ethics oversight—particularly if the “experiment” does not fit the format of a randomised controlled trial.

While there is clear legislation in most jurisdictions regarding research on laboratory animals and human patients, laws and standards for clinical research on veterinary patients are not well defined [81]. This means that veterinary clinical studies may lack oversight by an ethics committee, which would otherwise ensure that the study meets scientific, ethical, quality and animal welfare standards [81]. Bertout and colleagues point out that ethical oversight of veterinary clinical studies is particularly challenging in private practice settings, where the availability of such a review is lacking: “as such, private veterinary hospitals sometimes face hurdles when initiating or conducting clinical studies and must rely on the ethical review conducted by other participating centres or the sponsor, convene their own review panel, or end up having to forgo an ethical review altogether” [81].

The latter is disadvantageous to those wishing to publish their research, as journals (including this one) require authors to confirm that their research has been approved by or exempted from ethical review by an animal ethics committee, or written ethical justification of their work using the 3Rs [82]. To capture veterinary clinical studies in private practice settings, professional veterinary organisations may be able to provide ethical review and oversight. For example, the Royal College of Veterinary Surgeons provides an Ethics Review Panel for “practice-based researchers, who may not normally have access to such through university or industry connections” [83].

Such measures may be helpful in the context of prospective studies intended for publication, but the requirement for their input may not be flagged in situations where publication is not an intended outcome. Furthermore, deliberations, which require time for preparation and convening of a committee, may not assist individual patients for whom delayed intervention would yield negative consequences. As described by veterinary ethicist Moses: “Only a small fraction of pet owners are able to afford hospital stays long enough to allow for someone to notice the ethical nature of a conflict, ask for a consultation, and have it done in the time frame during which decisions must be made” [84]. Getting the balance right between the provision of timely ethical oversight, and the ability of clinicians to exercise clinical judgement, may be challenging.

All veterinary treatment requires informed owner consent. In the case of experimental treatment, in addition to information about the potential risks of proceeding, as well as the evidence base (or lack thereof) for the treatment when compared to alternatives if these are available, owners should be informed that a treatment is experimental. This may be difficult, as some owners may cling to the hope, however unjustified, that an experiment will extend the life of their companion animal. The first author, as a staff member in a veterinary school who also works with companion animals, regularly receives unsolicited queries from owners of companion animals with life-limiting conditions seeking information about potential clinical trials they could enrol their animals in for this very reason.

## 7. How Can We Address Ethical Concerns Associated with Advanced Veterinary Care?

Thus far, we have outlined concerns about AVC identified in the veterinary ethics literature, including poor quality of life or negative impacts on animal welfare, dysthanasia, increased caregiver burden, financial costs and impacts on accessibility, conflicts of interest and a lack of oversight for what amounts to experimentation. These concerns are not exclusive to AVC, but may be exacerbated in the context of AVC, for example due to its potential to be more invasive or more costly. Despite these concerns, we believe that individual animals, their owners and veterinary professionals and practices can benefit from improved treatment of companion animals through improved animal welfare, an enhanced human–animal bond, professional development, compassion satisfaction, and income. However, the concerns raised demonstrate that AVC, simply by virtue of being “advanced”, is not enough to ensure “good” practice. Ethical AVC requires prospective and retrospective ethical review and thoughtful implementation.

The use of ethical frameworks, for example utilitarianism, the ethical matrix, and the four principles of biomedical ethics, prompt structured reflection on the potential harms relative to the potential benefits of healthcare [85,86]. For example, according to the first two principles of biomedical ethics, non-maleficence and beneficence [85], veterinary professionals should aim to minimise harms and maximise benefits associated with veterinary care. Indeed, Bley employs these principles as the basis of guidelines for clinical decision making in veterinary oncology [87]. According to this model, if treatment promotes a patient’s basic needs and interests or increases wellbeing, and the frequency, durational and intensity of side effects is outweighed by these, the treatment can be justified on ethical grounds.

Fraser’s “practical” ethic for animals requires that we provide good lives for animals in our care, treat suffering with compassion, be mindful of unseen or unintended harms and protect the life sustaining processes and balances of nature [88,89]. According to this framework, it is important that veterinary care is compatible with a good life (or a life worth living [90], or positive welfare [91]), aims to minimise suffering, has minimal negative unintended consequences, and is practiced sustainably. The latter tends to be considered in relation to livestock, but is rarely raised in relation to companion animals [92,93]. While it is possible that AVC may result in negative impacts in the environment, for example through the use of inhalational anaesthetics [94] or cytotoxic chemotherapeutic agents [95], we did not find authors raising specific concerns about AVC in this regard. Indeed, there is scope for AVC practitioners such as specialist anaesthetists to develop and promote sustainable practices. For example, advanced monitoring equipment may permit a reduction in inhalational anaesthetic use and associated greenhouse gases [94]. There is a need for further studies regarding the potential harms of all types of veterinary practice to the environment, and effective mitigation strategies [96]. In the future, environmental sustainability may be seen as a feature of AVC, and veterinary practice in general.

Fraser’s “practical” ethic ensures that the interests of the companion animal are central to ethical deliberation, emphasising the need to promote positive welfare and treat suffering with compassion. However, it is important that secondary interests are identified and appropriately managed. The Vet Ethics Tool, developed by Grimm and colleagues, was developed in response to concerns raised by the Association of Veterinary Anaesthetists (AVA) regarding “apparent inappropriate overtreatment of some companion animals” [49]. In aiding ethical deliberation around a proposed treatment, the tool requires users to consider primary factors, notably the interests of the animal, potential immediate and long-term harms and benefits of the proposed treatment, as well as risk and harm mitigation strategies. It also requires the consideration of secondary factors, including the experience of the team, the quality of the evidence on which the proposed treatment is based, the potential impact on the client (including the financial impact) and their relationship with the animal, the ability of the client to provide suitable aftercare, and a final priority check (whether secondary factors outweigh primary factors). The tool employs a traffic light system, with red indicating that alternative treatment options should be considered; orange indicating a need to reconsider the procedure and/or clinician’s responsibility, and green indicating valid justifications for the proposed treatment [49]. Importantly, the tool does not and cannot establish where the line is drawn between acceptable and unacceptable treatment. However, if applied conscientiously, it ensures a comprehensive assessment of primary and secondary justifications for a proposed intervention. Furthermore, it may be a useful tool in stimulating discussion among veterinary team members and may reduce the risk of interventions that lead to poor QOL or dysthanasia. The use of ethical frameworks and tools such as the Vet Ethics Tool in clinical settings may improve the ability of veterinary team members to recognise and manage conflicts of interest. The inclusion of a person or persons from outside of the organisation in these deliberations may reduce organisational bias.

In the light of concerns about companion animals being subjected to essentially unregulated experiments in some situations, as part of these deliberations, humane endpoints should be established and agreed upon in advance of commencing treatment, and owner consent appropriately documented [97]. This discussion should also include information about the potential caregiver burden associated with treatments [50]. In addition, discussion of proposed clinical trials or novel interventions should be undertaken by an ethics committee applying the 3Rs framework to help ensure that alternative treatments are considered, that an appropriate number of animals are enrolled in the study, and that methods are refined to minimise harms [98]. It is critical that negative or unexpected outcomes are published, as this can reduce the risk of flawed approaches being re-attempted [79].

Additionally, regulatory bodies may determine that some procedures should not be performed at all or should only be performed where stringent conditions are met. For example, the RCVS currently “does not support the use of living source donors for feline renal transplantation” because removal of a kidney from the source cat involves inflicting pain and discomfort which does not benefit that animal (27.33) [99]. Where dead animals are used as source animals, the animals must not have been euthanised for the purpose of donation (27.37); the owner of the source animal must provide informed consent (27.38), and centres which perform such procedures must consult with an ethics committee that includes a layperson (27.41e). In addition, the team performing the procedure must include veterinarians with Diplomate or Board certification in medicine, soft tissue surgery and anaesthesia, or microvascular surgery and critical care (27.41a), ensuring an appropriate skill set.

The use of QOL scoring tools, as well as pain scoring tools, needs to be normalised in veterinary clinical settings as well as in peer-reviewed companion animal studies, to provide baseline data and facilitate evaluation of the impact of AVC on the welfare of animal patients. The routine use of such instruments could improve the assessment of animal welfare in companion animal practice settings and may increase awareness of both veterinary team members and animal owners of the welfare of animals. Recording scores in electronic patient records may assist in the inclusion of patient welfare in retrospective studies. Ideally, owners should be able to access the same tools for QOL and pain scoring in companion animals, as there may be differences in scoring between veterinary team members and animal owners [100].

Formal clinical audit, currently utilised sporadically in veterinary settings, requires evaluating outcomes against explicit criteria with the goal of improving and refining practice [101]. Clinical audit should be cyclical, to ensure continuous improvement. Informal clinical audit may occur in the form of morbidity and mortality rounds [102,103]. The focus of the latter is typically adverse or unexpected outcomes in the morbidity and mortality rounds [102]. Such discussions may be broadened to incorporate ethical aspects of cases. Alternatively, ethics rounds may be helpful in identifying and alleviating moral distress among veterinary team members [84,104,105] and refining future practice. The authors of this study are conducting a pilot study to determine the impacts of virtual ethics rounds on veterinary team members.

Discussions around costs of AVC highlight disparities in access to veterinary care, and the need for a “safety net” for animals [57]. This underscores the need for veterinarians to be equipped to provide a spectrum of care, which in turn requires adequate exposure to general practice and primary care, as well as specialist or referral settings [12,13]. It has been argued that AVC should be accompanied by approaches that enable more clients to pay for it [16]. While strategies such as pet insurance can be helpful, premiums are not affordable for all pet owners. The availability of a spectrum of care is required to ensure that the welfare needs of the majority of companion animals can be met.

## 8. Conclusions

The veterinary ethics literature raises a number of ethical concerns regarding the AVC of companion animals. Awareness of these concerns, and the application of ethical frameworks and tools may aid in the reduction of harms and maximisation of benefits of AVC. Routine QOL assessment of veterinary patients in clinical settings and inclusion of QOL assessment in publications will aid evaluation and refinement of veterinary interventions including AVC. Clinical audit and ethics rounds may help to identify and alleviate distress among veterinary team members and may help refine AVC.

The provision of AVC highlights disparities in access to veterinary care and underscores the need for the availability of a spectrum of care.
